# Supplementary material for: Droplet-based high-throughput single microbe RNA sequencing by smRandom-seq
Source: Nat Commun. 2023 Aug 23;14:5130. doi: 10.1038/s41467-023-40137-9 (PMC10447461; doi:10.1038/s41467-023-40137-9)
Supplement: Supplementary file 1 — Supplementary information [file 41467_2023_40137_MOESM1_ESM.pdf]

Supplementary Information

Droplet-based high-throughput single microbe RNA sequencing by smRandom-seq

Ziye Xu[1,2#], Yuting Wang[2,3#], Kuanwei Sheng[4,5#*], Raoul Rosenthal[6], Nan Liu[2], Xiaoting Hua[7], Tianyu Zhang[2], Jiaye Chen[8], Mengdi Song[2], Yuexiao Lv[2], Shunji Zhang[3], Yingjuan Huang[2], Zhaolun Wang[2], Ting Cao[1,4,6], Yifei Shen[1], Yan Jiang[7], Yunsong Yu[7], Yu Chen[1], Guoji Guo[2], Peng Yin[4,5*], David A. Weitz[4,6*], Yongcheng Wang[1,2,3,4*]

[1]Department of Laboratory Medicine, the First Affiliated Hospital, Zhejiang University School of Medicine, Hangzhou, China

[2]Liangzhu Laboratory, Zhejiang University, Hangzhou, China

[3]College of Biomedical Engineering and Instrument Science, Zhejiang University, Hangzhou, China

[4]Wyss Institute for Biologically Inspired Engineering, Harvard University, Boston, United States

[5]Department of Systems Biology, Harvard Medical School, Boston, United States

[6]John A. Paulson School of Engineering and Applied Sciences and Department of Physics, Harvard University, Cambridge, United States

[7]Department of Infectious Diseases, Sir Run Run Shaw Hospital, Zhejiang University School of Medicine, Hangzhou, China

[8]Department of Biomedical Informatics, Harvard Medical School, Boston, United States

#These authors contributed equally to this work

*Corresponding Author: Yongcheng Wang (yongcheng@zju.edu.cn), David A. Weitz (weitz@seas.harvard.edu), Peng Yin (Peng_Yin@hms.harvard.edu), and Kuanwei Sheng (Kuanwei.Sheng@wyss.harvard.edu)

Supplementary Figures

Supplementary Figure 1

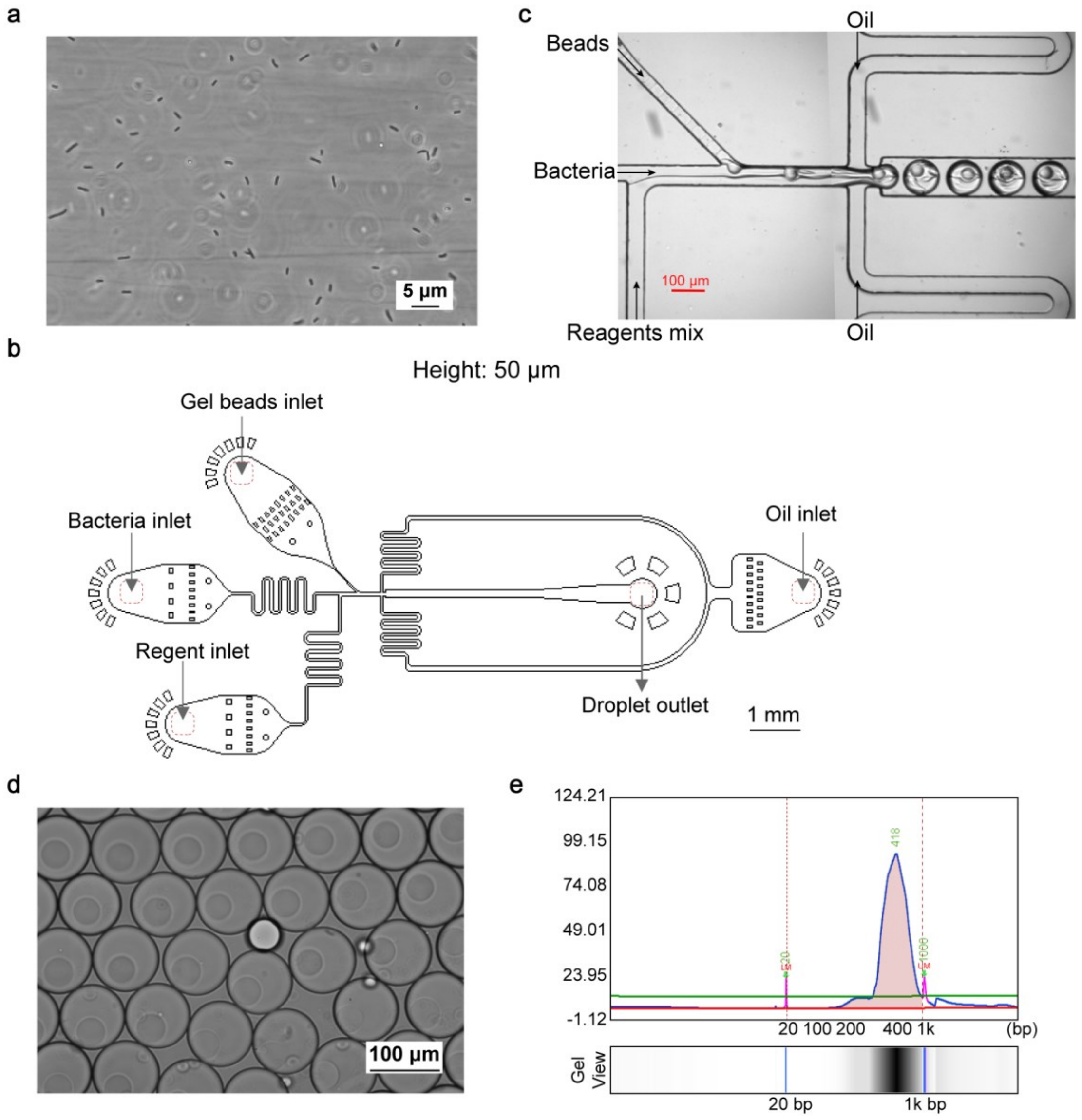

Supplementary Figure 1. The microfluidic platform of smRandom-seq.

a, Image of fixed *E. coli* after reverse transcription and dA tailing. Scale bar: 5 μm. n= 4 independent experiments. **b**, Design of the device for cell, bead and mix reagents encapsulation. Height: 50 μm. **c**, Image of microfluidic barcoding device. Scale bar: 100 μm. **d**, Image of encapsulated droplets. Scale bar: 100 μm. **e**, Electropherogram of amplicon from barcoded single bacterium cDNAs. Lower marker: 20 bp; upper marker: 1k bp.

Supplementary Figure 2

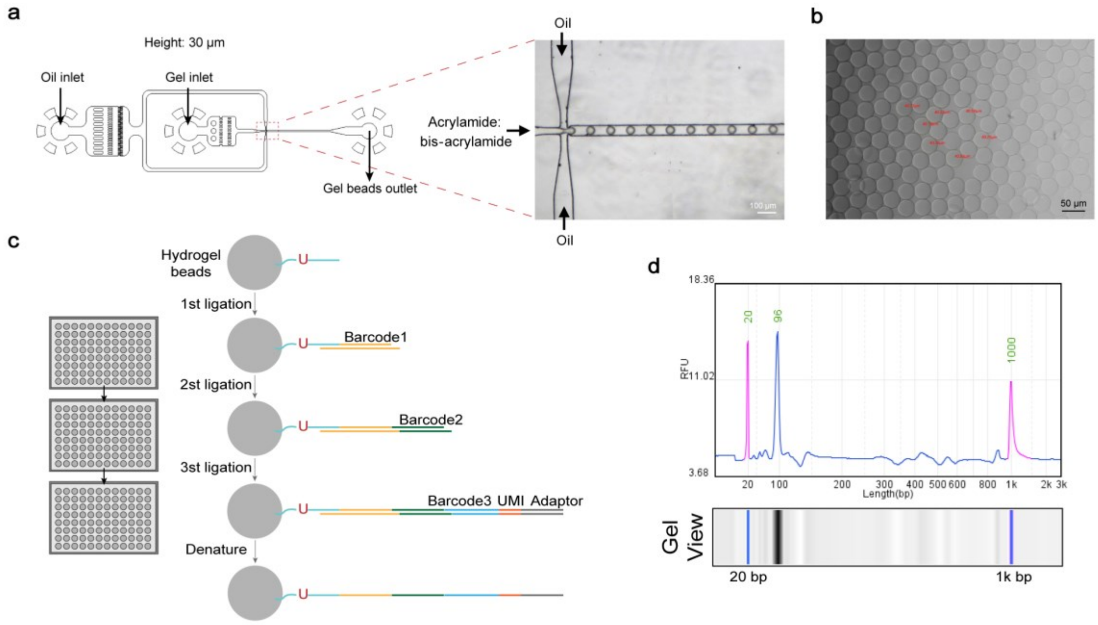

Supplementary Figure 2. Synthesis and quality control of barcoded hydrogel bead.

a, Design of the device for hydrogel beads generation. Height: 30 µm. Hydrogel bead generation and collection. Scale bar: 100 µm. **b**, Image of hydrogel beads. Scale bar: 50 µm. **c**, Barcode beads fabrication procedures. U: uracil residue; UMI, unique molecular identifier. **d**, Electropherogram of released DNA primers after enzymic digestion from barcode beads. Peaks at 20 and 1000 base pairs are gel migration markers.

Supplementary Figure 3

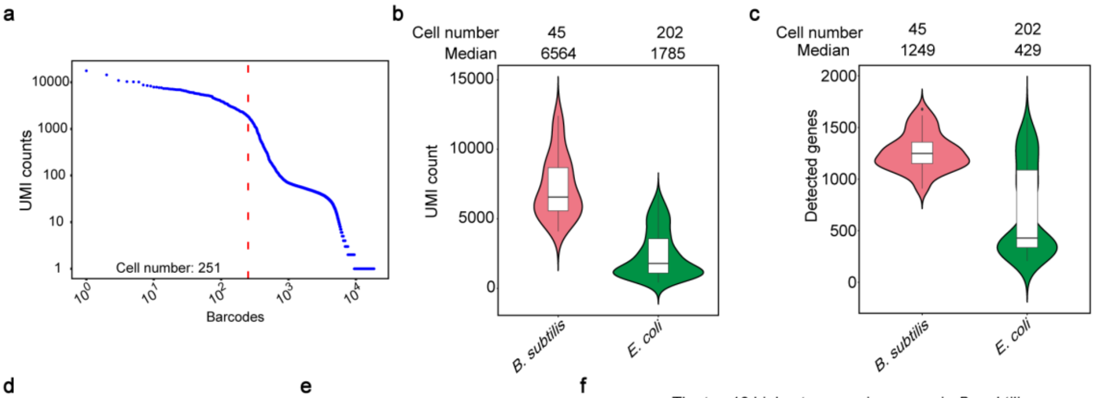

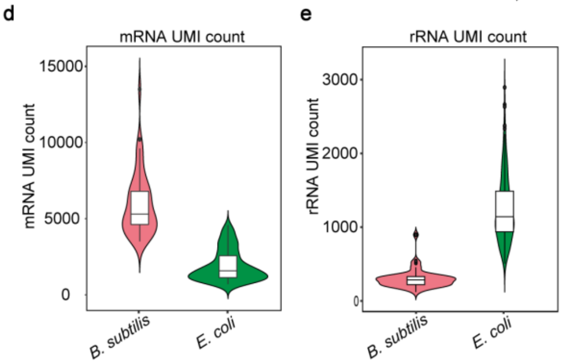

f

The top 10 highest expression genes in *B. subtilis*.

smRandom -seq		Public bulk RNA -seq	
**Gene ID**	**Count proportion**	**Gene ID**	**Count proportion**
B4U62_00715	0.032357487	B4U62_00710	0.021309632
B4U62_00710	0.024757256	B4U62_00715	0.020266442
B4U62_21090	0.017682124	B4U62_00870	0.009370901
B4U62_00745	0.015061197	B4U62_03460	0.009284874
B4U62_16755	0.012081833	B4U62_00830	0.009189792
B4U62_00870	0.00932849	B4U62_21090	0.008860777
B4U62_00485	0.009104753	B4U62_02035	0.008415549
B4U62_03370	0.00902028	B4U62_00745	0.008308393
B4U62_00700	0.00899745	B4U62_10560	0.007835396
B4U62_19050	0.008785127	B4U62_00690	0.007555582

Supplementary Figure 3. Performance of the smRandom-seq on reference bacteria samples. **a**, UMI count versus barcode rank plot of the *E. coli* and *B. subtilis* mixture. The knee point (red dashed line) indicates the threshold for *E. coli* and *B. subtilis* mixture (Cell number: 251). **b**, **c**, Distribution of UMI counts (**b**) and detected genes (**c**) from *B. subtilis* and *E. coli* in the mixture of *B. subtilis* and *E. coli* sample by smRandom-seq without rRNA depletion. *B. subtilis* n= 45 cells, *E. coli* n= 202 cells. **d**, **e**, Distribution of mRNA UMI counts (**d**) and rRNA UMI (**e**) counts detected from *B. subtilis* and *E. coli* in the mixture of *B. subtilis* and *E. coli* sample by smRandom-seq without rRNA depletion. *B. subtilis* n= 45 cells, *E. coli* n= 202 cells. **f**, The top 10 highest expression genes sorted by the count proportion in *B. subtilis* datasets of the species mixture by smRandom-seq and the public bulk RNA-seq dataset of the Emp3h_i Exponential growth sample[1]. Data in the box plot in **Supplementary Fig. 3b-e** corresponded to the first quartiles (lower hinges), median (center), and third quartiles (upper hinges). Whisker is defined as 1.5* IQR (interquartile range) from the hinge.

Supplementary Figure 4

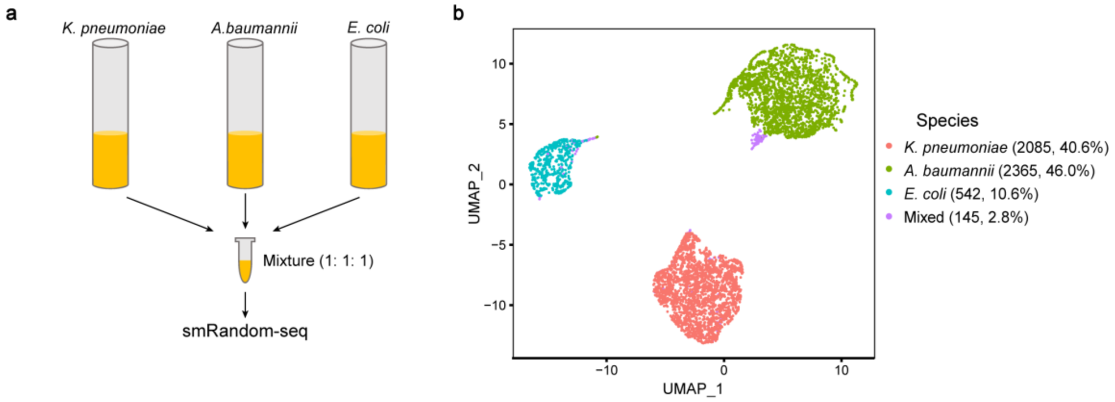

Supplementary Figure 4. Three-species-mixing experiment.

a, Experimental design for three-species mixture including *A.baumannii*, *K. pneumoniae*, and *E. coli* by smRandom-seq. **b**, UMAP plot colored by species, *A.baumannii*, *K. pneumoniae*, *E. coli* and mixed cells, identified *from the* three-species mixture.

Supplementary Figure 5

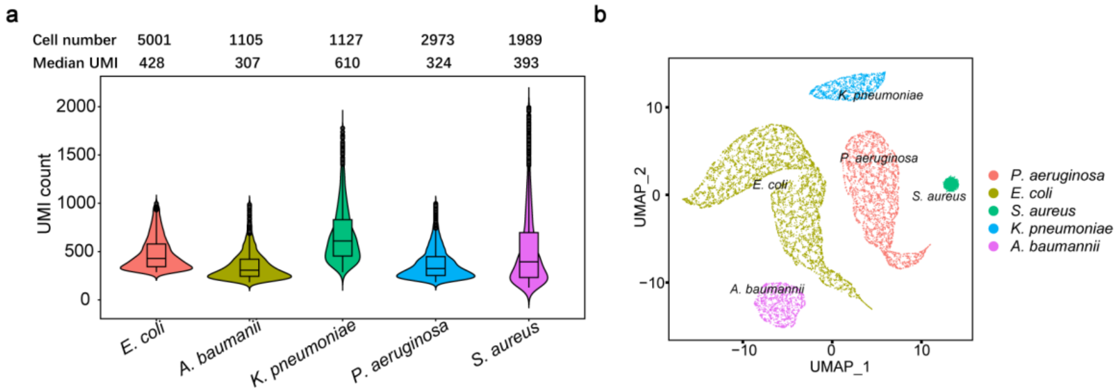

Supplementary Figure 5. Single-species experiments.

a, Distribution of UMI count of different bacterial species datasets by smRandom-seq, including *E. coli*, *A. baumannii*, *K. pneumoniae*, *P. aeruginosa*, and *S. aureus*. The cell numbers of these bacterial species datasets by smRandom-seq were as follows: 5001 *E. coli*, 1105 *A. baumannii*, 1127 *K. pneumoniae*, 2973 *P. aeruginosa*, and 1989 *S. aureus*. Data in the box plot in **Supplementary Fig. 5a** corresponded to the first quartiles (lower hinges), median (center), and third quartiles (upper hinges). Whisker is defined as 1.5* IQR (interquartile range) from the hinge.

b, UMAP plot colored by species, *E. coli*, *A. baumannii*, *K. pneumoniae*, *P. aeruginos*, and *S. aureus*, identified from single-species experiments. Source data are provided as a Source Data file.

Supplementary Figure 6

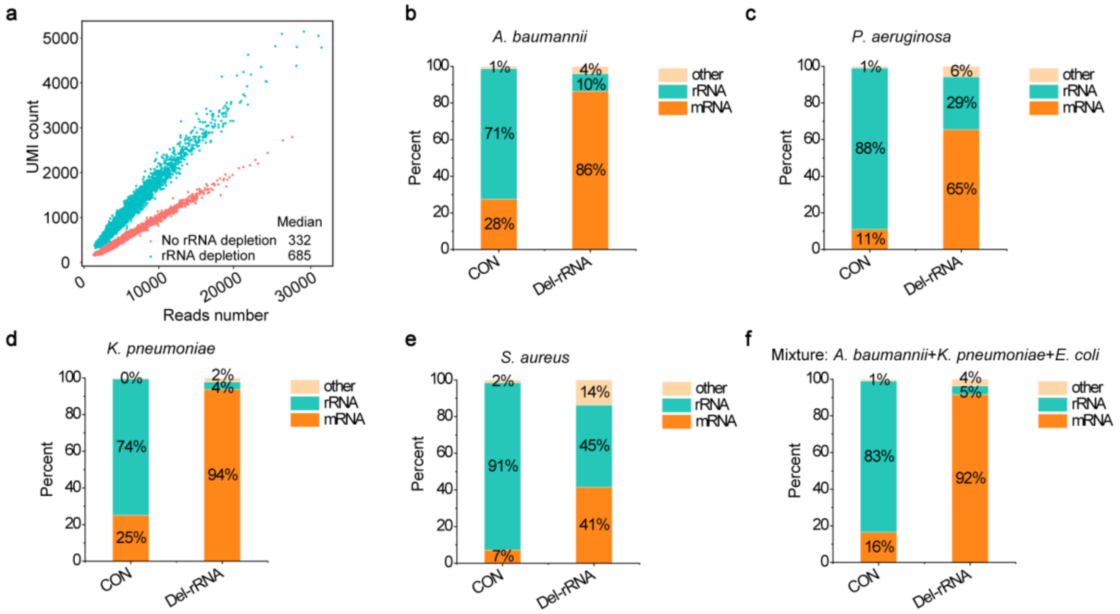

Supplementary Figure 6. Case9-based rRNA depletion experiments.

a, Scatter plot showing UMI count and the number of reads per barcode of *E. coli* samples with (blue) and without (red) rRNA depletion. Each dot corresponds to a putative cell. **b-f**, Representative proportions of transcript categories of single bacterial species, including *A. baumannii* (**b**), *K. pneumoniae* (**c**), *P. aeruginosa* (**d**), and *S. aureus* (**e**), and a mixture of *A. baumannii*, *K. pneumoniae*, and *E. coli* (**f**) with (Del-rRNA) and without rRNA depletion (CON). other, all other RNA classes. Source data are provided as a Source Data file.

Supplementary Figure 7

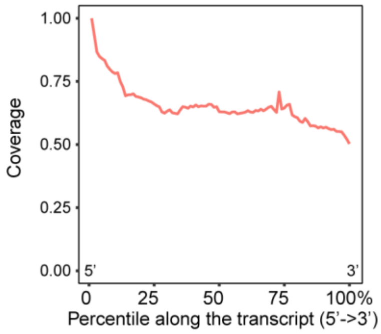

Supplementary Figure 7. Average percentile coverage across all transcripts (5'->3'). Source data are provided as a Source Data file.

Supplementary Figure 8

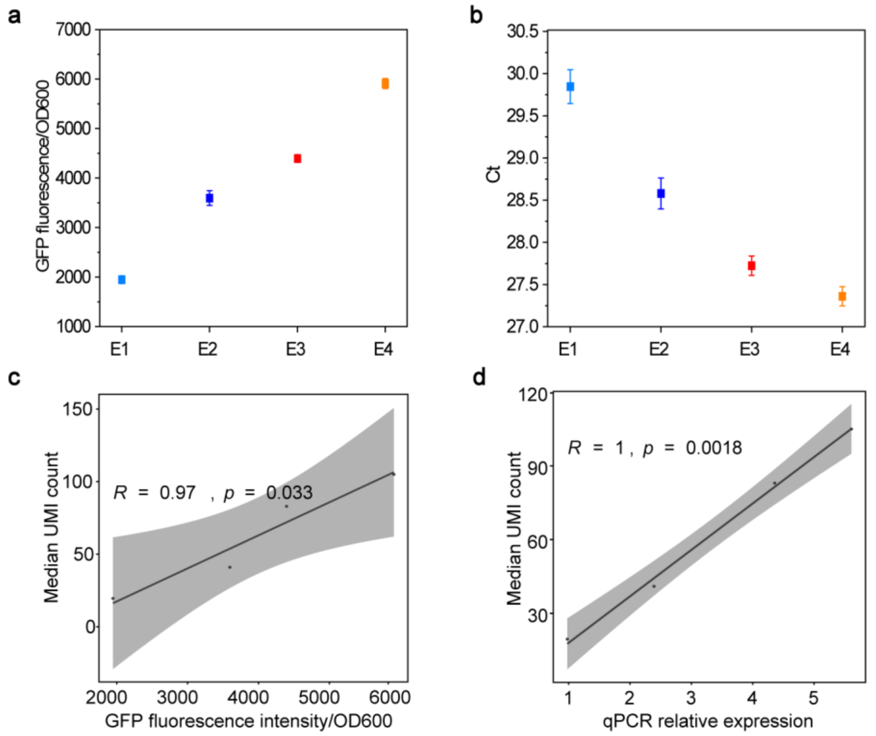

Supplementary Figure 8. Quantitativeness of gene expression in smRandom-seq by GFP expression model.

a, **b** GFP fluorescence/OD600 values (**a**) (n=6) and Ct values from qPCR (n=3) (**b**) of *E. coli* samples with different GFP expression induced by different amount of propionate (0, 6.25, 25, and 100 mM). Data in **a**, **b** were expressed as mean ± SEM. **c**, Comparison of GFP fluorescence values and the median number of UMIs. **d**, Comparison qPCR relative expression and the median number of UMIs. Relative quantification in qPCR is calculated by comparing the expression levels of GFP in other groups (E2, E3 and E4) against the expression levels of GFP in control (E1). *R* presents Pearson correlation coefficient. The shaded area represents the 95% confidence interval. *p*-value is the significance level of the *t* test and calculated alongside the Pearson coefficient as implemented in the R package ggscatter. Source data are provided as a Source Data file.

Supplementary Figure 9

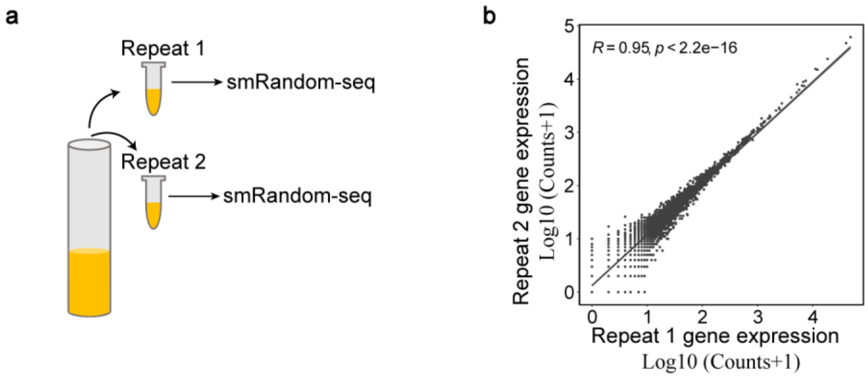

Supplementary Figure 9. Evaluation the technical reproducibility of smRandom-seq.

a, Experimental design for technical reproducibility evaluation. **b**, Scatter plot showing correlation of gene expression (Log10(Counts+1)) for two repeated *E. coli* samples (Repeat 1 and Repeat 2) applied with smRandom-seq separately. *R* presents Pearson correlation coefficient. *p*-value is the significance level of the *t* test and calculated alongside the Pearson coefficient as implemented in the R package ggscatter. Source data are provided as a Source Data file.

Supplementary Figure 10

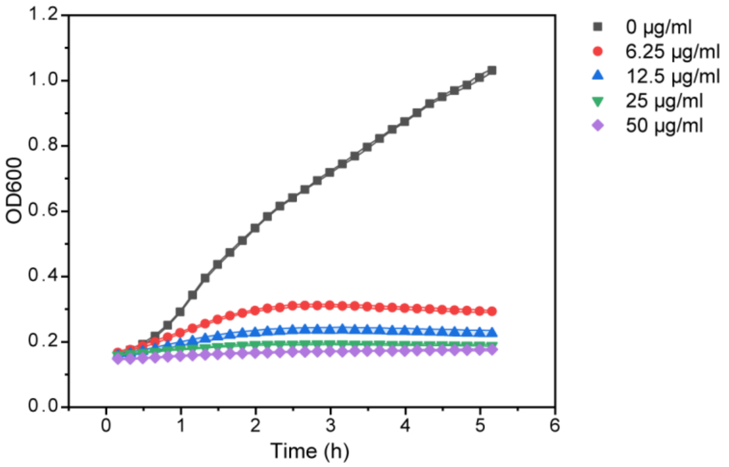

Supplementary Figure 10. Growth curves (OD 600) of *E. coli* upon 0, 6.25, 12.5, 25, and 50 μg/mL CIP. CIP: ciprofloxacin. Source data are provided as a Source Data file.

Supplementary Figure 11

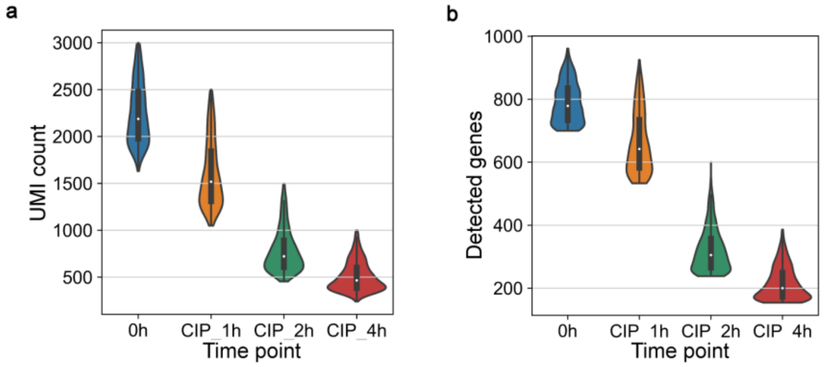

c

The top 10 highest expression genes in *E. coli*.

CIP-T0 by smRandom-seq		Species-mixture by smRandom-seq		Public bulk RNA-seq	
**Gene ID**	**Count proportion**	**Gene ID**	**Count proportion**	**Gene ID**	**Count proportion**
BW25113_RS19530	0.1785308	BW25113_RS19995	0.145887	BW25113_RS19530	0.30111
BW25113_RS01010	0.149809035	BW25113_RS19530	0.066561	BW25113_RS19995	0.211552
BW25113_RS23140	0.101970708	BW25113_RS01025	0.060744	BW25113_RS01025	0.211342
BW25113_RS13535	0.073189522	BW25113_RS19520	0.043218	BW25113_RS13535	0.064297
BW25113_RS01025	0.065432498	BW25113_RS13535	0.042552	BW25113_RS19520	0.058034
BW25113_RS23125	0.055954742	BW25113_RS04685	0.012936	BW25113_RS19980	0.039734
BW25113_RS19995	0.041493869	BW25113_RS17310	0.012513	BW25113_RS17000	0.038523
BW25113_RS13525	0.036868505	BW25113_RS01010	0.012182	BW25113_RS16985	0.029103
BW25113_RS22750	0.030024506	BW25113_RS09310	0.011086	BW25113_RS01010	0.024043
BW25113_RS16985	0.027230109	BW25113_RS00570	0.009709	BW25113_RS23125	0.00807

Supplementary Figure 11. Gene expression of CIP-treated *E. coli* samples.

a, b, Violin plots of the distribution of UMI count (**a**) and detected genes (**b**) of CIP-treated *E. coli* samples. 0h n= 771 cells, CIP 1h n= 850 cells, CIP 2h n= 950 cells, CIP 4h n= 949 cells. Data in the violin plot in **Supplementary fig. 11a, b** corresponded to the median of the distribution (white dot in the center), the interquartile range of the data (the thicker black bar), and the data that extends to 1.5 times the interquartile range (the thinner black bar). **c**, The top 10 highest expression genes sorted by the count proportion in *E. coli* datasets of the CIP-T0 sample, the species mixture by smRandom-seq and the public bulk RNA-seq dataset of the BW25113 Wild Type_ECWT1_1 sample[2].

Supplementary Figure 12

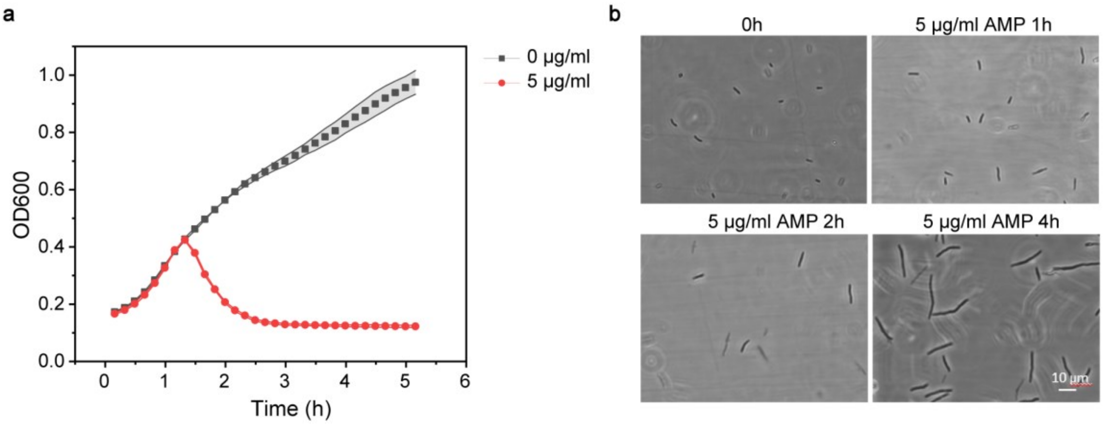

Supplementary Figure 12. Growth curves and morphology of *E. coli* upon AMP.

a, OD600 curves of *E. coli* exposure to 0 and 5 μg/mL AMP. Source data are provided as a Source Data file. **b**, Image of *E. coli* samples treated with 5 μg/mL AMP after 0, 1, 2, and 4 hours. Scale bar: 10 μm. n= 4 independent experiments.

Supplementary Figure 13

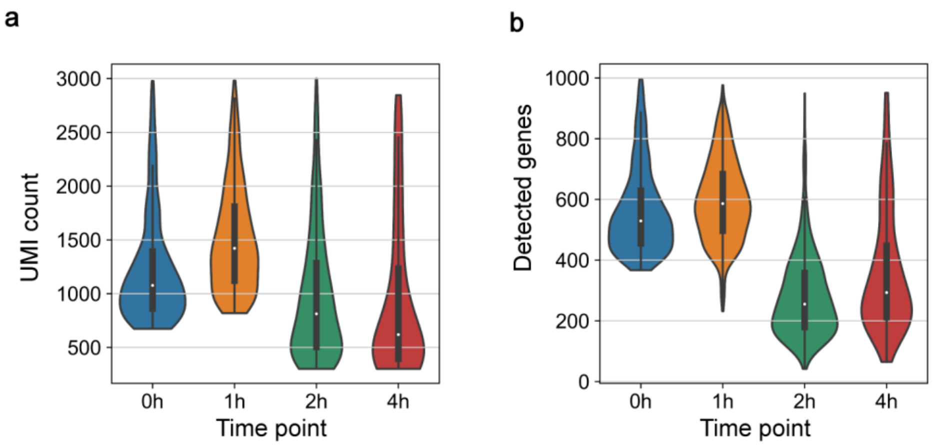

Supplementary Figure 13. UMI count (a) and detected genes (b) of AMP-treated *E. coli* samples. 0h n= 831 cells, AMP 1h n= 1094 cells, AMP 2h n= 716 cells, AMP 4h n= 216 cells. Data in the violin plot in **Supplementary fig. 13a, b** corresponded to the median of the distribution (white dot in the center), the interquartile range of the data (the thicker black bar), and the data that extends to 1.5 times the interquartile range (the thinner black bar).

Supplementary Figure 14

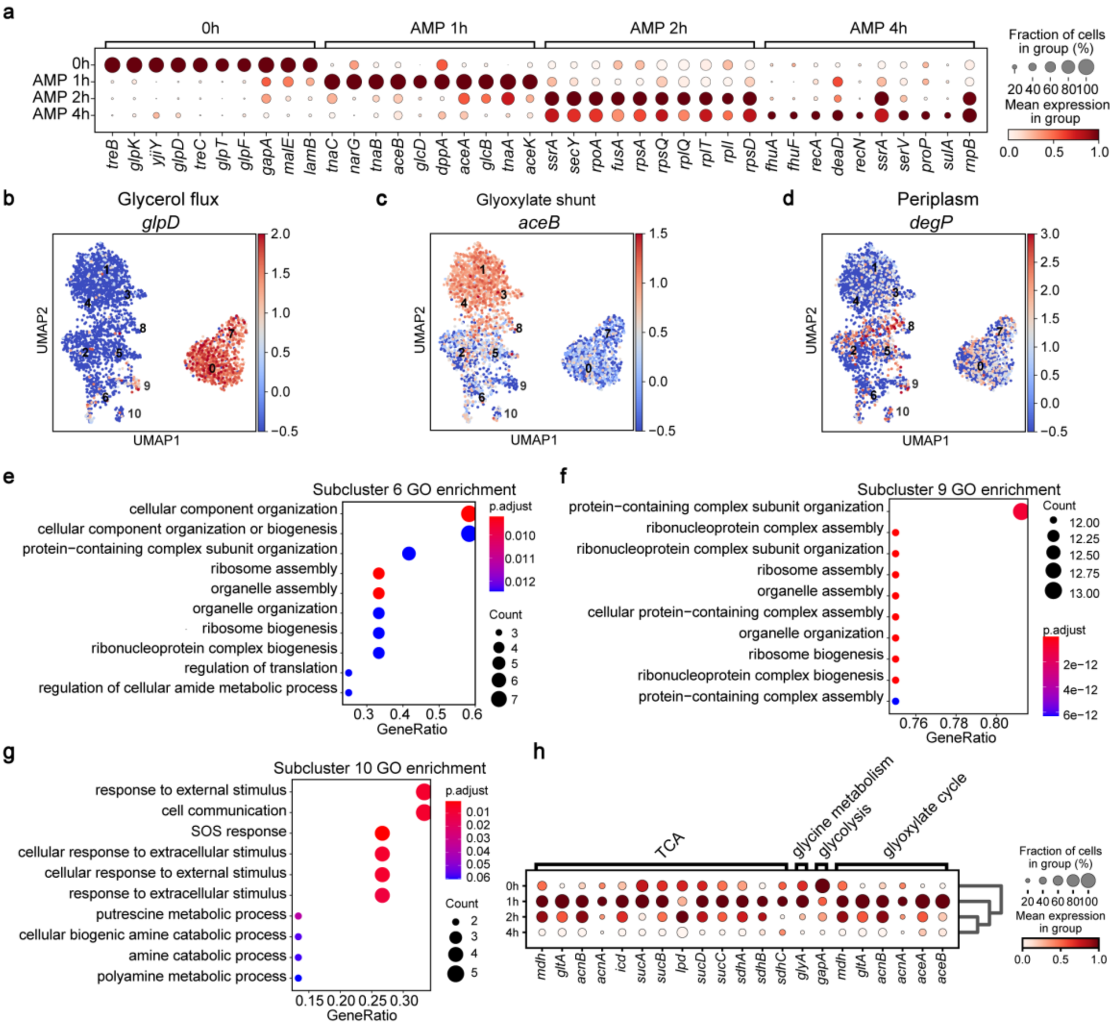

Supplementary Figure 14. Gene expression patterns of *E. coli* upon 12.5 μg/mL AMP.

a, Top 10 DEGs among groups of *E. coli* samples upon 12.5 μg/mL AMP. **b**, Expression of glycerol flux related marker genes of subclusters 0 and 7 (*glpD*) on the t-SNE plot. **c**, Expression of glyoxylates shunt related marker genes of subclusters 1, 3 and 4 (*aceB*) on the t-SNE plot. **d**, Expression of periplasm related marker genes of subclusters 8 (*degP*) on the t-SNE plot. **e-g**, Bubble plots of GO enrichment analysis of top 20 DEGs in subclusters 6 (**e**), 9 (**f**) and 10 (**g**) of *E. coli* samples upon high concentration of AMP. The cutoff value of p-value of GO enrichment was 0.05 and the cutoff value of q-value of GO enrichment was 0.05. **h**, Expression of genes involved in different metabolic pathways in different groups. TCA: tricarboxylic acid cycle. Source data are provided as a Source Data file.

Supplementary Figure 15

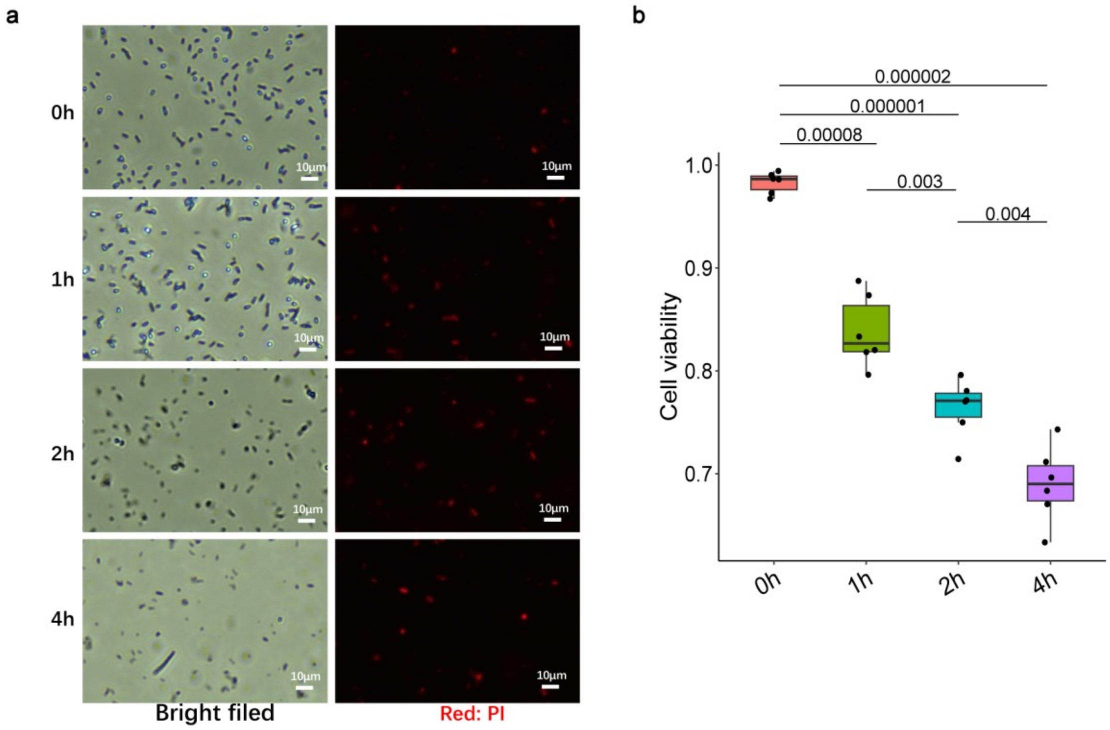

Supplementary Figure 15. Cell viability analysis of *E. coli* upon 12.5 μg/mL AMP.

a, Cell viability analysis by combined propidium iodide (PI) staining. Representative fluorescence and bright filed images of *E. coli* samples at 0h, 1h, 2h, 4h time point after 12.5 μg/mL AMP treatment. Red color (PI) showed positive staining of dead cells. Scale bar showed 10 μm. n= 4 independent experiments. **b**, Graphic representation of statistical result of cell viability. n= 6 biologically independent samples. Data in the box plot corresponded to the first quartiles (lower hinges), median (center), and third quartiles (upper hinges). Whisker is defined as 1.5* IQR (interquartile range) from the hinge. Two-tailed Student's *t* test were used for calculation of *p* values. Statistical significance was established at 95% confidence level (p values < 0.05). Source data are provided as a Source Data file.

Supplementary Figure 16

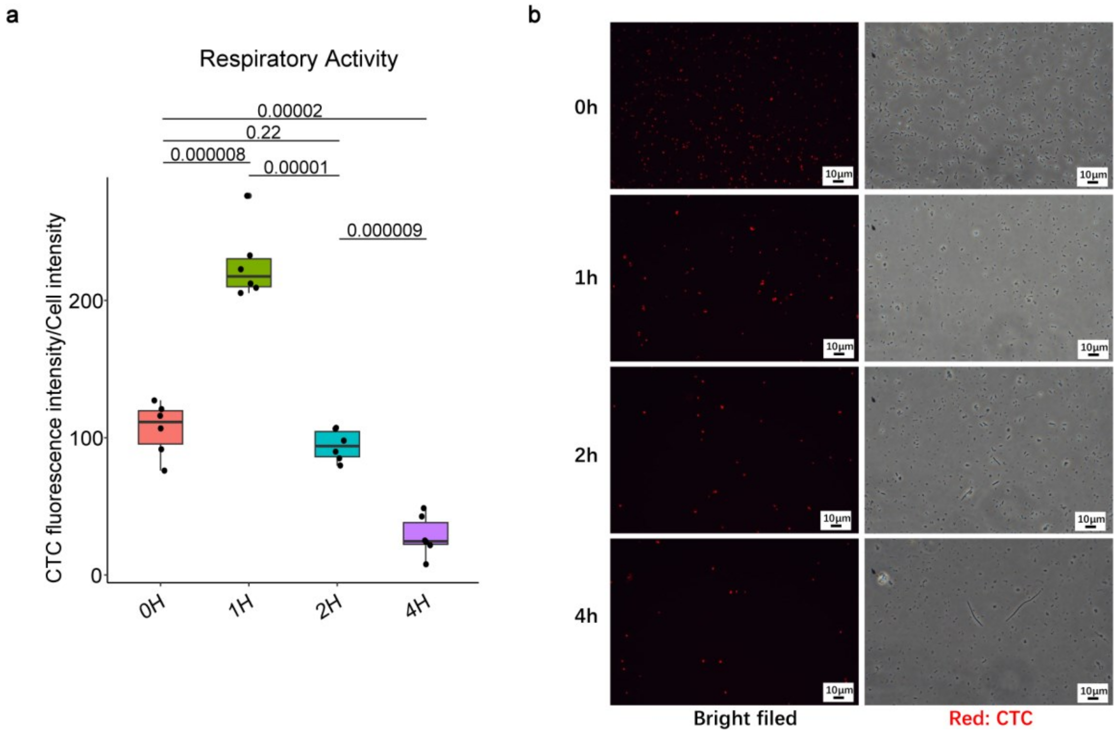

Supplementary Figure 16. Cell respiratory activity analysis of *E. coli* upon 12.5 μg/mL AMP. **a**, Cell respiratory activity analysis by the 5-cyano-2,3-ditolyltetrazolium chloride (CTC) dye staining in combination with fluorescence microplate reader. Graphic representation of statistical result of normalized CTC fluorescence intensity (CTC fluorescence intensity/Cell count) of *E. coli* samples at 0h, 1h, 2h, 4h time point after 12.5 μg/mL AMP treatment. n= 6 biologically independent samples. Data in the box plot corresponded to the first quartiles (lower hinges), median (center), and third quartiles (upper hinges). Whisker is defined as 1.5* IQR (interquartile range) from the hinge. Two-tailed Student's *t* test were used for calculation of *p* values. Statistical significance was established at 95% confidence level (p values < 0.05). **b**, Representative fluorescence and bright filed images of *E. coli* samples at 0h, 1h, 2h, 4h time point after 12.5 μg/mL AMP treatment. Red color (CTC) showed cells with high cell respiratory activity. Scale bar showed 10 μm. n= 4 independent experiments. Source data are provided as a Source Data file.

Supplementary Tables

Supplementary Table 1: Primers

Primer list	Sequence
Barcoded beads primer	/5Acryd/ATTATATATAT U GTG AGT GAT GGT TGA GGA TGT GTG GAGATA [10 bases barcode1] TGGT [10 bases barcode2] GAGA [10 bases barcode3] NNNNNNNNTTTTTTTTTTTTTTTTTTTT
Random primer	GGAGTTGGAGTGAGTGGATGAGT GATG NNNNNNN
PCR-1 primer	GGAGTTGGAGTGAGTGGATGAGTGATG
PCR-2 primer	GTG AGT GAT GGT TGA GGA TGT GTG GAG ATA

Supplementary Table 2: Barcode sequences

	Barcode1_sequences	Barcode2_sequences	Barcode3_sequences
1	GATGTACATG	ATAGAAACGA	GCTTTTCTTG
2	TTCAGATTCT	TCCGTGCAAG	CTTTAAGCAT
3	CGTACTACGC	AATGGGACAC	GAAATATTGT
4	GCTATTATAG	TATTAGGCGA	GGCGGAAACG
5	CAGAAGGAAC	CAATCATCAC	AATGGGTATG
6	AGACTTTTAA	CAGTTTTACT	CGGCAATTGA
7	GGCAACTGTG	CTTTCTAGAT	CTCGAGGAAT
8	AAATTCGCGG	TGTGATACCT	CCTATTGTCT
9	CTTAGACACT	TGTTAACAAC	GAGGCTCCTA
10	AGCGGACTAT	TTGCTTTCAG	ACTCAAGGGA
11	GAGACGCTAC	CAGGAACTAA	ACATCCTGCT
12	GTCATCGTGC	TCGGGCGGAT	ATTATGGTAG
13	TCATTAACAG	ACGTATAGTG	TACCGGATTA
14	GCTTCGCTTA	TACTTAAGGC	CAATTTGAAC
15	CTGGCAAAGA	CATGCGGGTG	TTTATAGACC
16	TCGCTTGAAG	TCGCACTCCG	ATCACTCGGT
17	ATGAACTTTG	GAAACTGAAA	ACTACTAATA
18	CAGAGATACG	TACAGGCGCA	CCGGAGGATA
19	GGCTGATAAA	GTTGGCATTT	GACGGGAGGT
20	TATTTGTCGC	GTTTTAGGAA	TCTGGAATAG
21	TTAAGGCAAA	AGATTGGCGG	TGGAGGGAGC
22	ACAGTATTAT	GAGCGGGTTT	GCTTGGCAAC
23	TGGATGCTAT	CGGGAAAGGG	GAGGAATCCT
24	TCGAATTTCG	TTCTTAGGCG	AAGTAAACGG
25	TACTATGGCG	AACACAATAA	TTTTGTGGTG
26	TAGCTTAGTG	TTCCGGCGAG	TCATTGATTG
27	GTCAGGCATG	TTTGTTTTAT	CAGTATCATC
28	GGTTGGTGGT	GCAGGTGCAG	CACATGGTGT
29	ACATGTAGCT	ATAAGGACCT	GTATAGAGTG
30	GCGAACAATG	TCAGTTATCC	TCGGGTAATC
31	TGTTGCTTGC	AATGGAATGT	AATTAAGAGC
32	TAACAGCAAT	TGTAATTGAT	TCACAGCACA
33	GTTTGGACTC	TCAGTGGGTT	AATTACCTCT
34	CATTATGCTA	GTGGACGTTG	TGTTCATAGA
35	GAGTAAGTGA	CTCCTGCAAA	CACGGTGTAG
36	GAGCGGTGTA	TTCGGATTCC	GCCGAGGTGT
37	CGTAAGTCGA	AATCGGGTCA	ATGAGGCTTT
38	TGGCACGTTT	AGTCGCCGAC	TGATGGCGGA
39	GAAATCTTTT	CATAAGAAGT	CGCTCCTGGT
40	AACAACGCAA	AGGCAGGAAA	TCAAGGCCTG
41	ATGTTGTGGC	CTTATCCTTG	ATTAAAGGCT
42	TAACTCATTC	GGTCGAAGGG	CGGAAAGAGT
43	TCGACTATAT	CAAGAGGTGA	ACAATAGGCG
44	TATCAGTCTG	CTACTGGTGG	TCCGACCGTC
45	CTATATGTAG	TATGTTCATC	GAAGATACAA
46	TGCAATACGG	AGGCTTGTTC	CTAAAGGACG
47	AAACTGGTGT	TCGACAGCAG	TATAAAGGAA
48	AATTAGGCGT	GTAAATGGGA	ACAGGAGTGA
49	ACTGTTCAGG	CGGTAATACG	
50	CAGTATTGTT	CATTAGTAAA	
51	GTACTATAGT	TTAAAGGTTC	
52	ACAGACATTT	TAGAAATGGT	
53	GTCTTTGGAT	CAACAATGGG	
54	CTTGATCTAT	AAATATCGAA	

55	GTATGTCAGG	GCCTGGTAGT	
56	TATTCAACTG	AAAGACTCTT	
57	TTTTGACGGA	TTAACACTTA	
58	ATGGTAGAAA	GTTAAGGCTA	
59	GTGCTCTATC	GTCACAGGTT	
60	GTTATAGTTG	TTTGAAAGTG	
61	AGGATCTTAC	GATTGGGTGC	
62	TGCATTTGAA	TAAAGGATGT	
63	AATTCGGATA	TATTCAATAT	
64	GGTTGAGAGC	GATGATCGAC	
65	CAAGGCTCAT	TAGTGGAGTC	
66	GAATGGAAGC	GCTAAACTTC	
67	CTTCTATATG	TTTTGATTAA	
68	TCTGGATTAC	ACTGTTTAGA	
69	TTGATCAGTA	ATGAGTGATA	
70	ATTTTTGTCG	CTATACCTCC	
71	TACGACTGAA	TCTTGGCTCA	
72	AAAGTTGCAA	CGACAGAATG	
73	GGCGTGCTTT	CCTCCTGGAG	
74	CGGCGGATTT	AGCATTTTCT	
75	CGACAAAGTG	TACTAAGTAG	
76	TGCGTAATTC	AGTTTAAGGA	
77	CAAATGTCGT	AAACTGGCCT	
78	CAGCTAGCTT	ATAGGTAGGG	
79	ACACGGCTCT	TATCAGGGTC	
80	ATTATGACTG	GAATGCGCAA	
81	ACGCATAGAC	CACGGGTCAA	
82	TGGAAGGACT	ATGGGTCGCC	
83	TAAGTAGATA	CGGATACAAA	
84	CACAAGTATA	TCGCCGGTTA	
85	TAAGAATGCG	TAAATGTAAA	
86	ATATGTGTGA	GCAGTGGAAA	
87	ACACAGTGTG	CGAGTAGTCT	
88	AGCACTGTAT	ATTTCTTCAA	
89	ACTATGCGAG	TGTTTGATCC	
90	CTACATGCTC	CGGGTCTATC	
91	CTTCTGGTGG	CTGCGGGATG	
92	GGATGCTCGA	GCGGTGCTCT	
93	CAGCGAAGGA	GTAACGCAGA	
94	GTAAAGACGT	TGATACAAAT	
95	TTGCTACAAC	CCTAGGTCAC	
96	TGGAAACGGT	TCTTTAGCGG	

Supplementary References

1. Wang B, K.F., Hamoen LW Induction of the CtsR regulon improves Xylanase production in Bacillus subtilis. *Preprint from Research Square* (2023).
2. Ojo, O., Scott, D., Iwalokun, B., Odetoyin, B. & Grove, A. Transcriptome RNA Sequencing Data Set of Differential Gene Expression in Escherichia coli BW25113 Wild-Type and slyA Mutant Strains. *Microbiol Resour Announc* **10** (2021).
