# Supplementary material for: Droplet-based high-throughput single microbe RNA sequencing by smRandom-seq
Source: Nat Commun. 2023 Aug 23;14:5130. doi: 10.1038/s41467-023-40137-9 (PMC10447461; doi:10.1038/s41467-023-40137-9)
Supplement: Supplementary file 3 — Description of Additional Supplementary Files [file 41467_2023_40137_MOESM3_ESM.pdf]

Title: Supplementary Data 1

Description: sgRNAs for rRNA depletion of smRandom-seq (**Fig. 2d**, **e**, **f**, **Supplementary Fig. 6**).

Title: Supplementary Data 2

Description: A summary of the datasets by smRandom-seq (**Fig. 2**, **3**, **4**)
