# Supplementary material for: Droplet-based high-throughput single microbe RNA sequencing by smRandom-seq
Source: Nat Commun. 2023 Aug 23;14:5130. doi: 10.1038/s41467-023-40137-9 (PMC10447461; doi:10.1038/s41467-023-40137-9)
Supplement: Supplementary file 6 — Reporting Summary [file 41467_2023_40137_MOESM6_ESM.pdf]

nature portfolio

Corresponding author(s): Dr. Yongcheng Wang, Dr. David A. Weitz, Dr. Peng Yin, Dr. Kuanwei Sheng

Last updated by author(s): Jun 28, 2023

Reporting Summary

Nature Portfolio wishes to improve the reproducibility of the work that we publish. This form provides structure for consistency and transparency in reporting. For further information on Nature Portfolio policies, see our Editorial Policies and the Editorial Policy Checklist.

Statistics

For all statistical analyses, confirm that the following items are present in the figure legend, table legend, main text, or Methods section.

n/a	Confirmed	
☐	☒	The exact sample size (*n*) for each experimental group/condition, given as a discrete number and unit of measurement
☐	☒	A statement on whether measurements were taken from distinct samples or whether the same sample was measured repeatedly
☐	☒	The statistical test(s) used AND whether they are one- or two-sided<br>*Only common tests should be described solely by name; describe more complex techniques in the Methods section.*
☒	☐	A description of all covariates tested
☐	☒	A description of any assumptions or corrections, such as tests of normality and adjustment for multiple comparisons
☐	☒	A full description of the statistical parameters including central tendency (e.g. means) or other basic estimates (e.g. regression coefficient) AND variation (e.g. standard deviation) or associated estimates of uncertainty (e.g. confidence intervals)
☐	☒	For null hypothesis testing, the test statistic (e.g. *F*, *t*, *r*) with confidence intervals, effect sizes, degrees of freedom and *P* value noted<br>*Give P values as exact values whenever suitable.*
☒	☐	For Bayesian analysis, information on the choice of priors and Markov chain Monte Carlo settings
☒	☐	For hierarchical and complex designs, identification of the appropriate level for tests and full reporting of outcomes
☐	☒	Estimates of effect sizes (e.g. Cohen's *d*, Pearson's *r*), indicating how they were calculated

Our web collection on statistics for biologists contains articles on many of the points above.

Software and code

Policy information about availability of computer code

Data collection	BGIMAGING CellView (x64, 4.11.20068.20211225) was used to acquire images.<br>Microplate Manager® 6 software (#168-9520) of xMark™ microplate absorbance spectrophotometer (Bio-Rad) was used to measure the optical density at 600 nm (OD600).<br>SpectraFluor Plus plate reader (Tecan) was used to measure GFP fluorescence.<br>Qubit 2.0 (Thermo Fisher Scientific) was used to quantitate the amount of DNA.<br>Q-Analyzer for Qsep100 (3.4.3.0.6593) was used to analyze DNA fragments.
Data analysis	1. Cutadapt (version 3.7) ws used to trim primer sequences and extra bases generated by the dA-tailing step.<br>2. STAR (2.7.10a) was used to perform genome mapping.featureCounts (2.0.1) and UMI-tools (1.1.2) were used to do gene annotation and counting.<br>3. scanpy (1.8.2) in python (3.9.7) was used to process count matrix, and do downstream analysis (UMAP, Differential gene expression).<br>4. ggpubr (0.4.0) in R(4.0.1) was used to do correlation analysis.<br>5. clusterProfiler (v3.16.0) in R(4.0.1) was used to do GO enrichment analysis.<br>6. OriginPro2017 (b9.4.0.220) and ggplot2 (3.3.5) in R(4.0.1) was used to generate plots.<br>7. All script files used in the analysis in this manuscript can be downloaded from GitHub at are available at https://github.com/wanglab2023/smRandom-seq.

For manuscripts utilizing custom algorithms or software that are central to the research but not yet described in published literature, software must be made available to editors and reviewers. We strongly encourage code deposition in a community repository (e.g. GitHub). See the Nature Portfolio guidelines for submitting code & software for further information.

Data

Policy information about availability of data

All manuscripts must include a data availability statement. This statement should provide the following information, where applicable:

- Accession codes, unique identifiers, or web links for publicly available datasets
- A description of any restrictions on data availability
- For clinical datasets or third party data, please ensure that the statement adheres to our policy

The smRandom-seq data generated in this study have been deposited in the Genome Sequence Archive database under accession code CRA011274 [https://ngdc.cncb.ac.cn/gsa/browse/CRA011274].

Human research participants

Policy information about studies involving human research participants and Sex and Gender in Research.

Reporting on sex and gender	not applicable
Population characteristics	not applicable
Recruitment	not applicable
Ethics oversight	not applicable

Note that full information on the approval of the study protocol must also be provided in the manuscript.

Field-specific reporting

Please select the one below that is the best fit for your research. If you are not sure, read the appropriate sections before making your selection.

☒ Life sciences ☐ Behavioural & social sciences ☐ Ecological, evolutionary & environmental sciences

For a reference copy of the document with all sections, see nature.com/documents/nr-reporting-summary-flat.pdf

Life sciences study design

All studies must disclose on these points even when the disclosure is negative.

Sample size	Sample size was determined based on similar studies in this field. For experiments involving quantification of GFP expression, the replicate number for GFP fluorescence/OD600 detection was 6, and the replicate number for qPCR experiment detection was 3. For Cell viability analysis and ell respiratory activity analysis, the the replicate number was 6.
Data exclusions	No data were excluded from analysis.
Replication	The microfluidic encapsulation experiment, beads synthesis experiment, and micrographs experiment were repeated at least four times independently with similar results. Validation experiments of smRandom-seq were repeated three times independently. Samples in antibiotic treatment experiments were collected ~1000 cells.
Randomization	The experiments were not randomized.
Blinding	The Investigators were not blinded to allocation during experiments and outcome assessment, because no bias could be made by the subject or the tester in the experiments performed.

Reporting for specific materials, systems and methods

We require information from authors about some types of materials, experimental systems and methods used in many studies. Here, indicate whether each material, system or method listed is relevant to your study. If you are not sure if a list item applies to your research, read the appropriate section before selecting a response.

Materials & experimental systems

n/a	Involved in the study
☒	☐ Antibodies
☒	☐ Eukaryotic cell lines
☒	☐ Palaeontology and archaeology
☒	☐ Animals and other organisms
☒	☐ Clinical data
☒	☐ Dual use research of concern

Methods

n/a	Involved in the study
☒	☐ ChIP-seq
☒	☐ Flow cytometry
☒	☐ MRI-based neuroimaging
